# Intracranial abscess formation in an adult alpaca: a case report

**DOI:** 10.1186/s12917-019-1930-9

**Published:** 2019-06-04

**Authors:** Sonja Franz, Sandra Högler, Michaela Gumpenberger, Agnes Dadak

**Affiliations:** 1 Clinic for Ruminants, Clinical Unit of Ruminant Medicine, University of Veterinary Medicine Vienna, Veterinärplatz 1, 1210 Vienna, Austria; 2 Institute of Pathology, University of Veterinary Medicine Vienna, Veterinärplatz 1, 1210 Vienna, Austria; 3 Clinical Unit of Diagnostic Imaging, University of Veterinary Medicine Vienna, Veterinärplatz 1, 1210 Vienna, Austria; 4 Institute of Pharmacology and Toxicology, University of Veterinary Medicine Vienna, Veterinärplatz 1, 1210 Vienna, Austria

**Keywords:** South American camelids, Brain, Abscess, Otitis, Computed tomography, Pathology, Neurologic disease

## Abstract

**Background:**

Intracranial abscess formation is an extremely rare and sporadically documented disease in South American Camelids (SACs). Herein we report the first case of otogenic brain abscess formation in this species.

**Case presentation:**

A 4 years old female alpaca was presented to our veterinary hospital with a 6 month history of neurologic disorder symptoms, mainly head tilt to the right and emaciation. A comprehensive workup (ultrasound and computed tomography) revealed irreversible cranial nerve abnormalities, extensive lesions in the region of external, middle and internal right ear including destruction of bony structures (tympanic bulla, parts of temporal bone) and severe brain deformation caused by an intracranial abscess. The lesion was up to 6x7x4 cm and occupying almost 40% of the cranial cavity. No pathological findings were evident in other organs or structures. The late referral of the alpaca at this advanced stage of destructive disease precluded surgical intervention.

**Conclusions:**

This case report describes the clinical signs, diagnostic procedures and pathological findings in an adult female alpaca suffering from cranial nerve abnormalities caused by a massive otogenic brain abscess. Camelids suffering from otitis may not present with clinical signs until the pathology is severe. The importance of considering intracranial abscess formation as differential diagnosis in SACs showing the merest hint of nerve deficits cannot be emphasized enough in order to diagnose such pathological processes at an early and treatable stage.

## Background

In South American camelids (SACs) neurologic disorders caused by infectious or non-infectious agents are not uncommon [1–3], albeit there are no epidemiologic studies published on the incidence of these diseases so far. Besides, only a few case reports are available providing insight into aetiology, diagnosis, treatment, and outcome of neurologic camelid patients [1–6].

In SACs brain abscesses causing neurologic symptoms usually occur in response to bacterial infections. They either develop through hematogenous spread of bacteria from other anatomical sites or they result from direct extension of cranial infections [2, 3]. Clinical signs can vary depending on the region of the brain affected. To the author’s knowledge, only three case reports are published on intracranial abscess formation in SACs [4–6]. Brain abscesses were mainly found in crias and described as being an important complication of failure of passive transfer. The formation of intracranial abscesses was reported once in a 2-week-old alpaca cria caused by *E. coli* [4] and another time in a 1-month-old alpaca cria caused by *Fusiformes* spp. infection [6]. A potential microbial dissemination originating from a molar abscess in the mandible was considered to be causative for development of brainstem and cerebellum abscessation in an 11 years old male alpaca [5].

To our knowledge, this is the first description of intracranial abscess formation in SACs occurring as a complication from otitis rather than hematogenous spread from another site of infection or from cranial infection. This case report describes the clinical signs, diagnostic procedures and pathological findings in an adult female alpaca suffering from cranial nerve abnormalities caused by a massive otogenic brain abscess.

## Case presentation

**Fig. 1:**
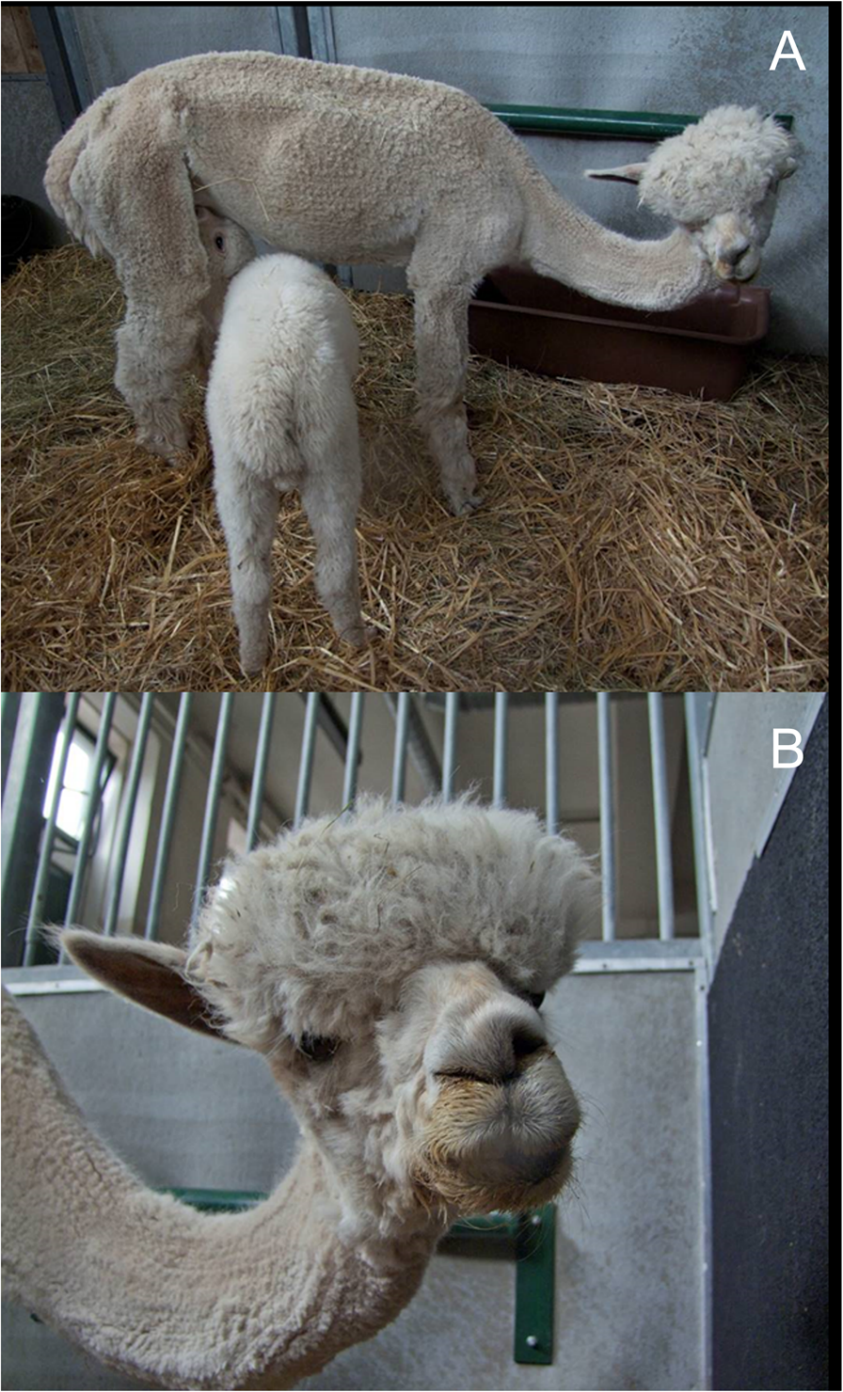
Adult female alpaca with its cria on initial physical examination. **a** the alpaca is emaciated and shows a lowered neck with head tilt to the right. **b** Collapse of the right nostril and ipsilateral lip droop

A 4 years old female alpaca was referred to the clinic for ruminants, University of Veterinary Medicine Vienna, Austria. The alpaca had a 6 month history of neurologic disorder signs. The main clinical signs were head tilt to the right and emaciation (Fig. 1a). The owner reported that he had treated the alpaca subcutaneously against mites several months before referral and that he had observed a slow deterioration of the clinical signs within the last 3 months. The alpaca separated from the herd but never showed inability to feed or dislike of the feeding. The alpaca was kept with other alpacas on a pasture, and all animals had access to additional feed such as hay and mineral supplements. No other alpacas on the farm were affected. The alpaca had given birth to a healthy cria 1 month before referral to the clinic.

On initial physical examination the female alpaca showed a quiet behavior, the body weight was 46.2 kg. Heart rate and respiratory rate were within reference ranges. The rectal temperature was 37.8 °C. Auscultation of lungs and heart was uneventful. No abnormalities were detected at abdominal palpation and auscultation. On both forelimbs the skin between the toes was hairless, thickened and crusty.

A neurologic examination was performed to determine the region of the nervous system being most likely affected.

Postural assessment of the alpaca revealed a lowered neck with a head tilt to the right and an ipsilateral lip droop with small amounts of cud dropping from the affected side. However, the animal was able to prehend food, chew and swallow. Further, collapse of the right nostril and deviation of the nasal philtrum to the left was observed (Fig. 1b). Atrophy of the muscles of mastication on the right side was diagnosed by palpation.

Additionally a plum-sized solid swelling at the base of the right ear was palpable. While the left ear was held in vertical position and moved constantly in response to sound, the right ear was held horizontally, never changing its position. No movement of the pinna occurred when testing the sensitivity of the right ear canal. There were no visible signs of trauma, alopecia or inflammation at the external pinna of the right ear.

In addition the alpaca showed loss of the menace reflex on the right side where the corneal as well as palpebral reflexes were impaired in contrast to the left side where immediate closure of the eyelid could be induced. Moreover, the alpaca was able to pass obstacles in a normal manner implying absence of significant visual impairment.

Observational gait analysis showed no ataxia or circling. Postural reaction (limb correction of awkward position) and spinal reflexes (withdrawal reflex, perineal reflex) were unremarkable.

In summary, neurological examination revealed multiple cranial nerve deficits. Dysfunction of the trigeminal nerve was linked to the observed atrophy of the masticatory muscle. Facial nerve defect was mainly responsible for pathological impaired corneal, palpebral and menace reflexes. Additionally, the defect of the facial nerve could have been causative for asymmetry of nose and lips, and even immobility of the right pinna, since no reflex could be induced by sensitivity testing. Dysfunction of the vestibulocochlear nerve resulted in head tilt.

Based on the neurological deficits identified by physical examination of this alpaca (cranial nerve deficits evident, no involvement of spine and limbs) the primary differential diagnosis included otitis media and interna.

For further diagnostic purposes, blood cell count was analyzed and showed a mild regenerative anemia (RBC 9.97 10^12^/L, Hb 6.5 mmol/L, MCV 31.9 fl, MCH 6.5 fmol/L, MCHC 20.5 mmol/L). Blood gas analysis and biochemistry profile were within reference ranges [7]. PCR analysis of whole blood was negative for Cand. *Mycoplasma haemolamae*. Parasitological examination of feces showed mild infection with *Coccidia* spp. Skin biopsies of the area between the toes of the forelimbs was tested positive for *Chorioptes* spp.

Otoscopic examination of the right external ear canal was performed, using a flexible endoscope with a diameter of 3.8 mm (Karl Storz Endoskop Austria GmbH). It was noticeable that the endoscope could be inserted into the external vertical ear canal for just about 0.5 cm, further insertion of the endoscope was not possible due to ear canal stenosis. The visible part of the external ear canal showed endoscopically no signs of inflammation and no pathological content (e.g. foreign body or parasites).

Ultrasound examination of the swelling at the base of the right ear was performed using a 7.5 MHz linear transducer and alcohol (ethanol 70%) as contact medium between probe and skin. At the area of interest a spheric inhomogenic hypoechogenic structure with small echoic spots was seen (loss of normal connective tissue and muscle architecture), sonographically resembling an inflamed tissue [8]. No cranial bone could be visualized in this area.

Analysis of cerebrospinal fluid (CSF) is one of the most commonly performed ancillary diagnostic tests when investigating the cause of a neurologic disease. Changes in protein concentration, cell count, and cell differentiation can help to distinguish between inflammatory/infectious and non inflammatory/non infectious diseases [2]. Nevertheless, in this case, CSF analysis was not considered, since physical examination implicated pathological changes of the middle and inner ear structures. Although there is limited information on the use of computed tomography (CT) to visualize brain and skull alterations in SACs [9, 10], the decision was made to perform a CT scan, since this imaging technique has been shown to be very effective in locating space-occupying lesions and pathological changes in brain tissue of other animal species.

**Fig. 2:**
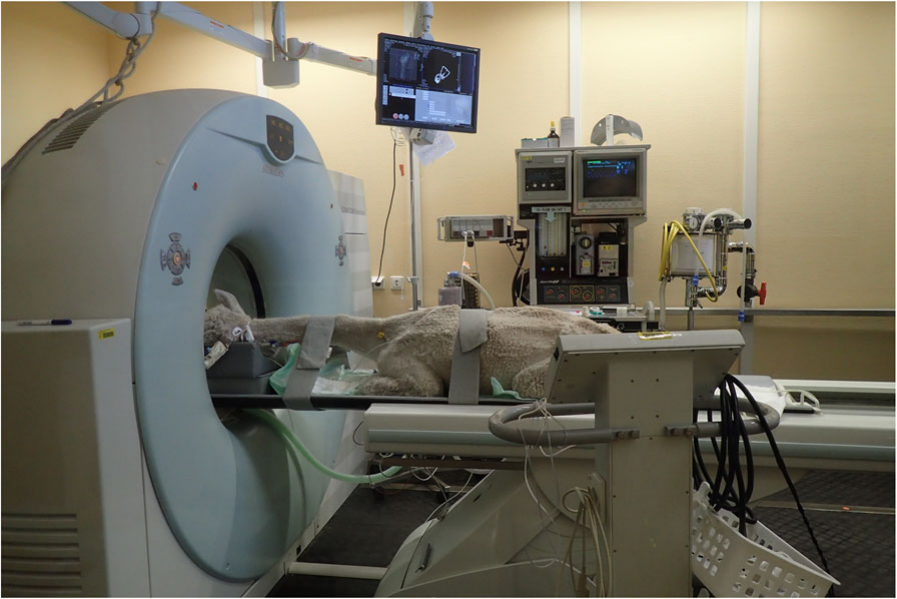
Positioning of the adult alpaca in sternal recumbency for CT examination of the head

CT examination (multi-slice helical CT, Siemens Somatom Emotion using 80 mAs, 130 kV, rotation time 1.5 s, pitch 0.8, and slice thickness 0.75 mm) took place at the Clinical Unit of diagnostic Imaging, University of Veterinary Medicine Vienna. The alpaca was examined under general anesthesia in sternal recumbency, using butorphanol (0.2 mg/kg IM) and xylazine (0.4 mg/kg IM) as premedication, followed by maintenance medication with ketamine (5 mg/kg IM) and isoflurane in oxygen (Fig. 2).

**Fig. 3:**
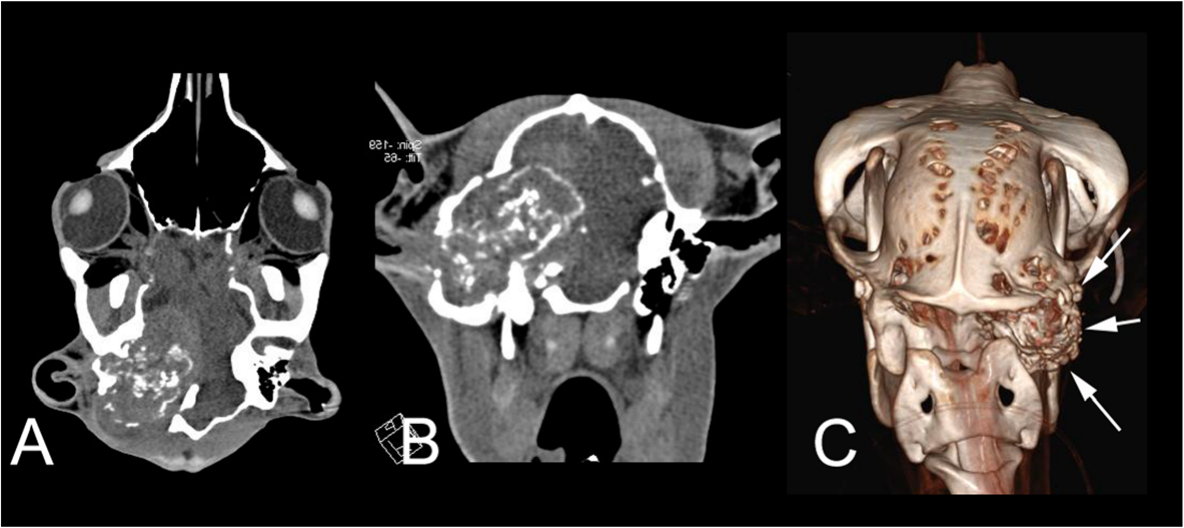
Coronal (**a**) and transverse (**b**) CT image of the head of the alpaca (soft tissue window). The right side of the animal is projected on the left side of the image. A partially mineralized soft-tissue mass originated at the level of the right ear and caused severe destruction of the temporal bone, midline shift of the brain and hydrocephalus (not seen on these planes). **c** shows a bony 3D-model of the head and first cervical vertebra of the same animal, caudal view

At CT examination (Fig. 3 a-c, 4A) the right osseous external ear canal was not apparent. A large space-occupying mass was replacing the right external acoustic meatus, and osseous ear canal, the area of the former tympanic bulla, tympanic cavity and inner ear. Remnants of the bulla wall were thickened and sclerotic. The ventral part of the tympanic bulla showed increased density with loss of all aerated areas of its usually honeycomb-like structure. The temporal bone showed thickening and intracranial irregular periosteal reactions dorsal to the right temporomandibular joint. The visible mass lesion was up to 6x7x4 cm (height x length x width) and was therefore occupying nearly 40% of the cranial cavity. The mass was mildly hyperintense with multiple, somehow onion shell-like calcified areas. No capsule was found. Both lateral cerebral ventricles were moderately enlarged, more on the right than on the left side. Marked midline-shift to the left was seen, causing complete compression of the caudal part of the right cerebral ventricle. The cerebellum was partially displaced into the great foramen of occipital bone resulting in a mild cerebellar herniation. Mild atrophy of the right temporal muscle was visible. The lymph nodes in the upper neck region were unremarkable.

Due to the poor prognosis, the owners decided to have the alpaca euthanized.

A full necropsy was performed at the Institute of Pathology, University of Veterinary Medicine Vienna. The skull was cut into coronary sections by a diamond saw. Samples of brain, skull and organs were fixed in 4% buffered formaldehyde solution. Samples containing bone were decalcified for 12 h in Decal® (quartett GmbH, Berlin, Germany). All samples were then embedded in paraffin wax. Sections of 1.5 μm thickness were cut and stained with hematoxylin and eosin (HE) for histological examination. Masson’s trichrome staining was performed to demonstrate collagenous fibers in the abscess capsule and Brown and Brenn staining was performed to detect gram positive and negative bacteria. A primary antibody against smooth muscle actin (SMA, mouse monoclonal antibody, no: M0815, dilution 1:1500, Agilent Technologies Österreich GmbH, Austria) was used to demonstrate vessels and a primary antibody against glial fibrillary acidic protein (GFAP, rabbit polyclonal antibody, no: Z0334, dilution 1:3000, Agilent Technologies Österreich GmbH, Austria) was used to identify astrocytes in brain sections by immunohistochemistry (IHC).

**Fig. 4:**
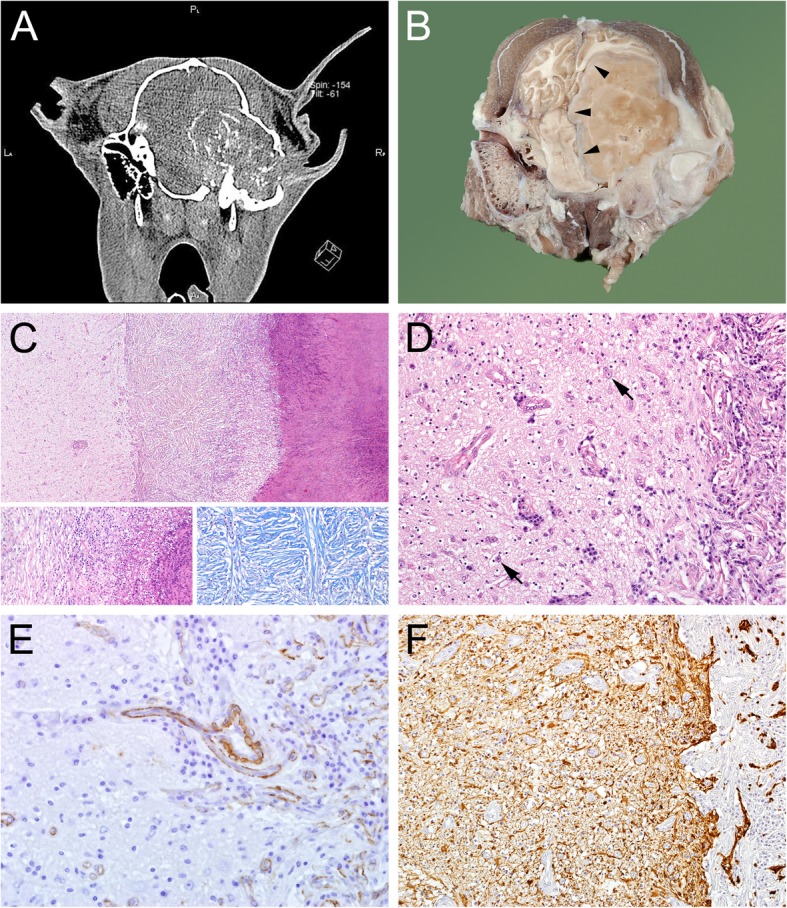
**a** CT image of the head of the adult alpaca (soft tissue window). A partially mineralized soft-tissue mass originated at the level of the right ear and caused severe destruction of the temporal bone, midline shift of the brain and hydrocephalus (not seen on these planes), **b** corresponding macroscopic section to A of the cranium on the height of the ear canal, arrowheads depict the thin capsule of fibrous tissue around the abscess; A + B: the left ear canal is clearly visible, while the right ear canal and cranium is replaced by the abscess, causing severe midline shift and displacement of the brain to the left. **c** histologic overview of the brain abscess. Neuropil in the left third, abscess capsule in the middle third, necrotic center in the right third of the picture (HE-staining). Bottom left insert: detail of abscess capsule with granulation tissue and collagenous fibers to the left, degenerated neutrophils and necrotic cell debris to the right (HE-staining). Bottom right insert: Collagenous fibers (dark blue) in the abscess capsule (Masson’s trichrome staining). **d** brain-abscess interface, abscess capsule to the right. Cerebral edema, gliosis and marked loss of neurons in the neuropil of the cerebral cortex, arrows: remaining neurons. Vascular proliferation and activation of endothelial cells and perivascular infiltration of lymphocytes (HE-staining). **e** brain-abscess interface, abscess capsule to the right. Infiltration of vessels from the abscess capsule into the neuropil (SMA-IHC). **f** severe diffuse astrogliosis (brown signal) in the neuropil of the cerebral cortex adjacent to the abscess capsule (GFAP-IHC)

On gross examination the animal was cachectic. An asymmetry of the skull was evident after the skin was removed. A mild swelling of firm consistency of the right base of the skull and discoloration and atrophy of the lateral portion of the right cerviculoscutular muscle were present. Coronary sections of the skull and brain showed a light brown-whitish gelatinous to firm mass of up to 7 cm in diameter replacing bone and muscle of the right cranium and expanding into the cranial cavity. The mass had a thin capsule of fibrous tissue (Fig. 4b). It encompassed the right middle and inner ear and occluded the right ear canal. The right tympanic bulla was completely destroyed in contrast to the normal left tympanic bulla (Fig. 4a, b). The mass caused severe midline shift in the caudal brain and displaced medulla oblongata, cerebellum, mesencephalon, and the caudal part of the right hemisphere to the left (Fig. 4b). The right hippocampus and thalamus were displaced cranially. A mild hydrocephalus of the lateral ventricles was evident due to obstruction of the mesencephalic duct.

Histologically the mass turned out to be an abscess with a great amount of necrotic debris in the center, followed by a layer of degenerated neutrophils adjacent to a capsule of granulation tissue and mature connective tissue (Fig. 4c). Mild to moderate infiltration of the outer layers of the capsule with lymphocytes and plasma cells was present and collagenous fibers were detectable by Masson’s trichrome staining (Fig. 4c). Within the necrotic debris degenerate bone fragments were present, while bone adjacent to the abscess capsule was resorbed by osteoclasts. Focally in the temporal bone, irregularly arranged osteoid was surrounded by osteoblasts and embedded in fibrous tissue adjacent to bone. Areas of bone resorption by osteoclasts were noted. In the neuropil adjacent to the abscess severe astrogliosis was detectable. Acute neuronal degeneration was not detectable, but regions with gliosis showed a marked loss of neurons (Fig. 4d). The neuropil was edematous and showed small foci of malacia in gray and adjacent white matter. Atrophy of the cerebral cortex by pressure was evident in the right occipital lobe directly adjacent to the abscess. In some regions vascular proliferation and infiltration of vessels from the abscess capsule into the neuropil as well as mild perivascular infiltration with lymphocytes were present (Fig. 4d, e). By immunohistochemistry severe diffuse astrogliosis was detectable in brain regions adjacent to the abscess (Fig. 4f). No bacteria were detectable histologically by Brown and Brenn staining in the brain abscess.

## Discussion and conclusions

In a patient suffering from a brain abscess of the size detected in this female alpaca a wider and more obvious range of clinical signs could have been expected than actually being observed in the presented case. Therefore, the importance of thorough physical and neurological examination in SACs showing the merest hint of nerve deficits cannot be emphasized enough in order to diagnose chronic processes at an early stage.

In general, differential diagnoses for cranial nerve deficits include otitis media/interna, trauma, listeriosis, aberrant parasite migration and brain abscess or tumor [1–4, 6].

Beside physical examination several different diagnostic approaches are available. To detect inflammatory processes of the CNS, the analysis of cerebrospinal fluid (CSF) in regard to pleocytosis and increased protein concentration is helpful [2, 3], but it needs to be considered that sampling of CSF requires sedation of the animal and is a risky procedure. In this case CSF analysis was not considered, since physical examination and the obvious cranial nerve deficits implicated pathological changes of the middle and inner ear structures which were confirmed by CT examination. In cases showing unspecific neurological clinical signs CSF analysis can provide useful diagnostic hints and therefore the authors would recommend this procedure in such patients.

Llamas and alpacas are predisposed for developing otitis media/interna because of characteristic anatomic conditions such as a long and narrow external acoustic ear canal and a multicompartmental tympanic bulla [3, 9, 11]. Otitis media/interna can arise from otitis externa or is often presumed to be a consequence of ascending infection up the eustachian tube [2, 3]. In the case presented, both ascending infection and otitis externa, could neither be confirmed nor ruled out as primary cause of the brain abscess due to the extensive lesions in the region of external, middle and internal ear structures and the chronicity of the process. However, due to the absence of pathological findings in other organs or structures otogenic origin seems very likely.

In general, bacteria, ectoparasites and foreign bodies, such as migrating plant awns have been described as causative agents for otitis externa, media and interna in SACs [3, 5, 11–14]. *Listeria monocytogenes* [13], *Actinomyces* spp., *Streptococcus* group D [14], *Nocardia* spp., and *Proprionibacterium* spp. have been isolated and associated with ear infections in SACs, but no consistent causative bacteria were described so far [11]. *Psoroptes* spp. is less common in SACs but primarily affects the ears [3, 15]. From this alpaca only *Chorioptes* spp. were isolated from skin scrapings of the forelimbs, but the owner reported a history of mite infestation and subcutaneous antiparasitic treatment months before the alpaca was referred to the clinic. At this time no mite species differentiation was performed. Unfortunately, a complete ear canal stenosis was evident in this alpaca preventing further diagnostic measures at this site including biopsies.

Diagnostic imaging techniques, such as CT or MRI (magnetic resonance imaging) can be very helpful for antemortem diagnosis and are advantageous compared to radiography in cases demanding the visualization of medial and inner ear structures and the brain [2, 9, 11, 16, 17]. In this case it facilitated a profound diagnosis including the size and location of the abscess and the subsequent prognosis.

The histological lesions present in this patient were consistent with lesions described in a study regarding the neuropathological progression of brain abscess formation in dogs [18]. The authors of this paper divided the evolution of brain abscess formation into four stages based on histological criteria. In chronic cases with capsule formation they found a necrotic center, a peripheral zone of inflammatory cells and fibroblasts, and a collagenous capsule. The abscess was organized in the same way in our case as revealed by the histological samples analysed, although the necrotic center was very extensive. The capsule was quite thin, but contained collagenous fibers demonstrated by Masson’s trichrome staining. This might be due to the long duration of abscess formation. Interestingly in our case a capsule could not be visualised by CT examination. It is known that in CT scans of individual patients the texture of the abscess can prevent differentiation between the central core of the abscess, the capsule and the surrounding brain tissue [16].

The formation of osteoid in the region of bone resorption was interpreted as callus formation and likely an attempt of tissue repair. Reactive astrocytes, gliosis and cerebral edema were also present in this alpaca as have been described in dogs [18]. The cerebral edema is most likely of vasogenic origin as a consequence of the physical breakdown of the blood brain barrier. Vascular proliferation and activation of endothelial cells was present in some regions of the neuropil adjacent to the abscess. Furthermore some vessels seemed to infiltrate the neuropil from the abscess capsule. This might be a reaction of the neuropil to the expansion and duration of the process. It also correlates to the lesions described in dogs with chronic brain abscesses, in which a layer of neovascularity in the periphery of the abscess associated with continuing inflammation of the surrounding neuropil was present [18]. As shown by immunohistochemistry activation of astrocytes and gliosis developed in the surrounding neuropil due to the chronicity of the process. This is a typical reaction pattern of the brain, which is generally found in areas surrounding severe focal lesions, areas responding to chronic neurodegenerative triggers or infections [19].

Based on the findings of gross examination of this alpaca an otitis can be assumed as primary disease in the case presented. As known from the case history mite infestation was detected and treated by the owner a prolonged time before referral. It can be hypothesized that in this alpaca chronic mite infestation of the external ear canal led to damage of the tympanic membrane and to secondary bacterial infection of the inner ear structures, and finally to brain abscessation.

In general, treatment of intracranial abscesses is difficult and involves parenteral administration of antimicrobial drugs such as penicillin, florfenicol or oxytetracycline, using high dosages in order to reach efficacy inside the abscess [3]. Often antimicrobial treatment alone is inadequate and a surgical approach has to be performed. Craniotomy with excision of a brain abscess was performed successfully in a 1 month old cria [6]. Nevertheless prognosis is described fair to guarded [3]. In this case craniotomy with excision of the abscess had not been feasible due to the huge size of the abscess, and even surgical drainage, a technique that is described for removal of abscesses adjoining the cranium [3, 6] would have failed due to size and gelatinous consistency.

To our knowledge this is the first case report describing the incidence of otogenic brain abscess formation in SACs, the clinical signs, diagnostic procedures and pathological findings.

## Data Availability

All data generated or analysed during this study are included in this published article.

## Abbreviations

CNS: Central nervous system
CSF: Cerebrospinal fluid
CT: Computed tomography
GFAP: Glial fibrillary acidic protein
HE: Hematoxylin and eosin
IHC: Immunohistochemistry
MRI: Magnetic resonance imaging
PCR: Polymerase chain reaction
SACs: South American camelids
SMA: Smooth muscle actin
